# Mobile Health Apps Providing Information on Drugs for Adult Emergency Care: Systematic Search on App Stores and Content Analysis

**DOI:** 10.2196/29985

**Published:** 2022-04-20

**Authors:** Sebastián García-Sánchez, Beatriz Somoza-Fernández, Ana de Lorenzo-Pinto, Cristina Ortega-Navarro, Ana Herranz-Alonso, María Sanjurjo

**Affiliations:** 1 Pharmacy Department, Instituto de Investigación Sanitaria Gregorio Marañón Hospital General Universitario Gregorio Marañón Madrid Spain

**Keywords:** emergency drugs, emergency medicine, emergency departments, emergency professionals, medication errors, drug characteristics, drug management, apps, mHealth, mobile health, digital health, smartphone, mobile phone

## Abstract

**Background:**

Drug-referencing apps are among the most frequently used by emergency health professionals. To date, no study has analyzed the quantity and quality of apps that provide information on emergency drugs.

**Objective:**

This study aimed to identify apps designed to assist emergency professionals in managing drugs and to describe and analyze their characteristics.

**Methods:**

We performed an observational, cross-sectional, descriptive study of apps that provide information on drugs for adult emergency care. The iOS and Android platforms were searched in February 2021. The apps were independently evaluated by 2 hospital clinical pharmacists. We analyzed developer affiliation, cost, updates, user ratings, and number of downloads. We also evaluated the main topic (emergency drugs or emergency medicine), the number of drugs described, the inclusion of bibliographic references, and the presence of the following drug information: commercial presentations, usual dosage, dose adjustment for renal failure, mechanism of action, therapeutic indications, contraindications, interactions with other medicinal products, use in pregnancy and breastfeeding, adverse reactions, method of preparation and administration, stability data, incompatibilities, identification of high-alert medications, positioning in treatment algorithms, information about medication reconciliation, and cost.

**Results:**

Overall, 49 apps were identified. Of these 49 apps, 32 (65%) were found on both digital platforms; 11 (22%) were available only for Android, and 6 (12%) were available only for iOS. In total, 41% (20/49) of the apps required payment (ranging from €0.59 [US $0.64] to €179.99 [US $196.10]) and 22% (11/49) of the apps were developed by non–health care professionals. The mean weighted user rating was 4.023 of 5 (SD 0.71). Overall, 45% (22/49) of the apps focused on emergency drugs, and 55% (27/49) focused on emergency medicine. More than half (29/47, 62%) did not include bibliographic references or had not been updated for more than a year (29/49, 59%). The median number of drugs was 66 (range 4 to >5000). Contraindications (26/47, 55%) and adverse reactions (24/47, 51%) were found in only half of the apps. Less than half of the apps addressed dose adjustment for renal failure (15/47, 32%), interactions (10/47, 21%), and use during pregnancy and breastfeeding (15/47, 32%). Only 6% (3/47) identified high-alert medications, and 2% (1/47) included information about medication reconciliation. Health-related developer, main topic, and greater amount of drug information were not statistically associated with higher user ratings (*P*=.99, *P*=.09, and *P*=.31, respectively).

**Conclusions:**

We provide a comprehensive review of apps with information on emergency drugs for adults. Information on authorship, drug characteristics, and bibliographic references is frequently scarce; therefore, we propose recommendations to consider when developing an app of these characteristics. Future efforts should be made to increase the regulation of drug-referencing apps and to conduct a more frequent and documented review of their clinical content.

## Introduction

### Background

Digital technologies are an increasingly relevant resource for health services because they can improve the quality, efficiency, and safety of health care, a particularly relevant issue in the event of emergencies, disasters, and other unplanned care situations [1]. In recent years, there has been a significant increase in the quantity and quality of mobile health apps owing to the efforts made by health professionals and app developers. At the beginning of 2021, almost 50,000 medical apps were available on the main download platforms (Apple App Store and Google Play Store) [2]. Mobile apps are changing the health care landscape because they facilitate the exchange of information among professionals, researchers, and patients and enable easy access to quality services during clinical practice [3,4].

The need for a quick response is one of the most prominent characteristics of emergency medicine. Examples of the high care burden experienced in emergency departments can be seen in the nearly 130 million visits in 2018 in the United States or the 30 million visits registered each year in Spain [5,6]. A variety of apps have been developed in recent years to improve patient care in these departments [7,8]. Medical emergency apps are now a key element of clinical practice as they can be used as clinical decision tools, case management tools, and sources of clinical information. A desirable feature of these apps is that they can be used quickly because of the need to provide a rapid response to the broad spectrum of clinical scenarios occurring in emergency departments. Recent studies on mobile devices and medical apps in emergency rooms [9,10] have shown that the apps most frequently used by emergency health professionals are medical formulary and drug-referencing apps (84.4%), followed by disease diagnosis and management apps (69.5%) [10].

Health care pressure, stressful situations, and the need for multiple high-alert medications make emergency departments the perfect setting for drug-related problems [7]. Insufficient information on drugs is the most common cause of medication errors, which can lead to adverse drug events involving temporary or permanent harm to patients and higher health care costs [11,12]. The information needed in an emergency department includes multiple drug characteristics such as indications, dosing, administration, pharmaceutical compatibilities, adverse reactions, interactions, and contraindications [11,13]. The usefulness of medical apps as a source of information on drug-related characteristics should be highlighted, although the literature still contains relevant gaps concerning these tools. To date, no study has addressed the quantity and quality of smartphone apps that provide information on emergency drugs.

### Objective

Therefore, the main objective of this study was to identify apps designed to assist health care professionals in managing drugs for adult emergency care and describe their main characteristics and functionalities. As secondary objectives, we designed a score to estimate the amount of drug information contained in each app and analyzed the relationship between this score and the relevant app characteristics. We also analyzed whether some of the variables selected could affect user satisfaction (app user ratings).

## Methods

### Search Strategy and App Selection

We performed an observational, cross-sectional, descriptive study of smartphone apps available on the iOS and Android platforms that provide information on drugs used for adult emergency care.

The methodology used for app selection was based on the PRISMA (Preferred Reporting Items for Systematic Reviews and Meta-Analyses) system [14]. To identify emergency drugs–related apps, a search was conducted between February 15 and February 19, 2021, on the digital distribution platforms Google Play Store (Android) and Apple App Store (iOS), which are the app stores with the most apps available at present [15]. The search terms were “emergency drugs*”* OR *“*fármacos de urgencias*”* and “emergency medicine*”* OR “medicina de urgencias y emergencias.*”* We extracted text from app store descriptions and selected apps available in English or Spanish whose content was fully dedicated to drugs commonly used in the emergency room (hereafter referred to as *emergency drugs apps*) and apps related to the field of emergency medicine that contained a section on medications (*emergency medicine apps*). Apps aimed at pediatric emergencies were excluded because of relevant differences in the use of drugs in children (eg, dosage, treatment algorithms, and selection). Both free and paid apps were included. Apps from the Google Play Store were downloaded onto a Xiaomi Mi 9 SE (version 9 PKQ1.181121.001; Android), and apps from the Apple App Store were downloaded onto an iPhone 11 (version 14.4; iOS).

### Ethical Considerations

No patients were involved in the study and therefore ethical board approval was not sought, as it is considered unnecessary under RD 1090/2015 regulating clinical trials with medicinal products and the Ethics Committees for Research with medicinal products, and Law 14/2007 on Biomedical Research.

### Data Extraction

We collected the following information from the download platforms: app name, operating system (Android, iOS, or both), developer affiliation, country of origin, language, category, cost, publication date, date of last update, size, version, number of downloads, and user ratings. These indicators are commonly used in studies on health-related apps [16-19]. The overall mean weighted user rating was calculated by considering the number of ratings from both app stores. For the rest of the analysis, when the same app was available on both platforms, we only considered the version available on the Google Play Store as Android is the leading operating system worldwide and the Apple App Store provides less information (no data on the number of downloads). Subsequently, all apps were downloaded and their contents were evaluated. We counted the number of drugs included in each app and determined whether they belonged to ≥1 drug classes. We then evaluated whether the apps contained information on the following fifteen drug-related characteristics: (1) commercial presentations, (2) usual dosage, (3) dose adjustment for renal failure, (4) mechanism of action, (5) therapeutic indications, (6) contraindications, (7) interaction with other medicinal products, (8) use in pregnancy and breastfeeding, (9) adverse reactions, (10) method of preparation and administration, (11) stability data and incompatibilities, (12) identification of high-alert medications, (13) positioning in treatment algorithms, (14) information about medication reconciliation, and (15) cost. The selection of these indicators was discussed by the research team based on the most frequent requests received from emergency medicine pharmacy services and drug information centers [12,13]. High-alert medications are defined as drugs that bear a heightened risk of causing significant patient harm when used erroneously [20]. Medication reconciliation is defined as the formal process in which health care professionals partner with patients to ensure accurate and complete medication information transfer at transitions of care [21].

We assigned a score of 0 to 15 according to the amount of drug information provided in the app. A score of 0 indicates that the app did not include any information about the 15 drug characteristics analyzed, and a score of 15 indicates that all characteristics were shown in the app. Finally, we also evaluated whether the apps included bibliographic references on drug-related concerns.

### Data Analysis

All apps were independently evaluated by 2 hospital clinical pharmacists (SGS and BSF). The variables were coded and entered in a Microsoft Excel spreadsheet. The Cohen κ coefficient was calculated using Reliability Calculator for 2 coders [22] to analyze the level of agreement between the data collected by each investigator. Following this analysis, disagreements on the reported results were resolved through iterative discussion and consensus.

A statistical analysis was performed using Stata (version IC-16; StataCorp). On the basis of previously published studies on mobile health apps, we measured the association between a series of app characteristics (developer, main topic, cost, and number of downloads) and user ratings (which indicate user satisfaction) or the score assigned to the app (which indicates the variety of content on drug information). We also analyzed whether the inclusion of bibliographic references could be influenced by the app developer (health-related or non–health-related). The Shapiro-Wilk test was used to evaluate whether continuous variables were normally distributed. For normally distributed data, differences were assessed using the 2-tailed Student *t* test for 2 categories and ANOVA for ≥2 categories; for nonnormally distributed data, the Mann-Whitney *U* test was used. The correlation between quantitative variables was evaluated using the Spearman correlation test. Categorical variables were compared using an uncorrected chi-square test or Fisher exact test, as appropriate. Statistical significance was set at *P*<.05.

## Results

### Mobile App Search

**Figure 1:**
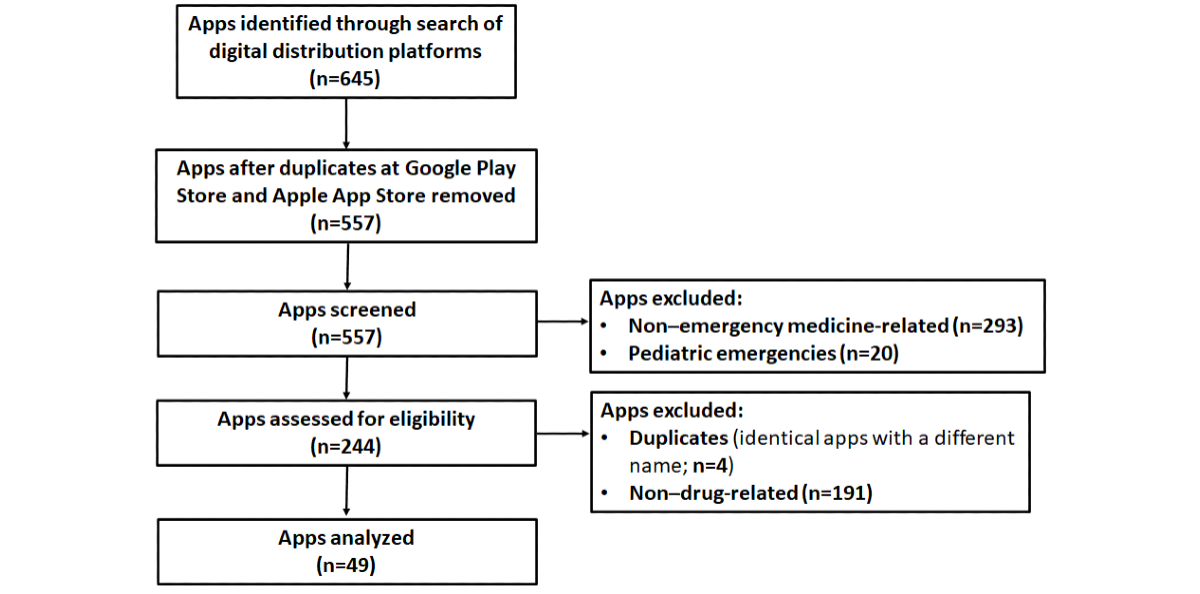
A flow diagram illustrating the search process for the apps analyzed in the study.

Combined keyword searches of the Google Play Store and Apple App Store yielded 645 apps potentially related to emergency drugs. A flow diagram illustrating the selection and exclusion of apps at various stages of the study is shown in Figure 1. We removed 88 apps as duplicates, with the same app name and developer appearing on both download platforms. The remaining 557 apps were further screened. We extracted information from the store app description and removed 293 apps that were not related to emergency medicine and 20 apps aimed at pediatric emergencies. We then exhaustively analyzed the descriptions of the remaining apps and downloaded them to determine whether the information was inaccurate. From the resulting apps, we removed 4 duplicates with different app names within the same store. We eventually excluded 191 apps that did not contain a specific section on drugs. Following this systematic search, we identified 49 apps that met the inclusion criteria. In total, 65% (32/49) of the apps were found on both digital distribution platforms, whereas 22% (11/49) were obtained only from the Google Play Store, and 12% (6/49) were only available from the Apple App Store.

Two independent researchers (SGS and BSF) further analyzed the characteristics, functionalities, and contents of the 49 apps selected. The mean Cohen κ coefficient for interrater reliability was 0.94 (SD 0.05).

### Analysis of General Characteristics of Apps

Textbox 1 shows the names of the 49 apps classified by their main topic (emergency drugs or emergency medicine).

By origin, 41% (20/49) of the apps were developed in North America, 35% (17/49) in Europe, 12% (6/49) in South America, 4% (2/49) in Asia, and 2% (1/49) in Africa. The origin of 6% (3/49) of the apps could not be determined. Of the 49 apps analyzed, 27 (55%) were published only in English, 21 (43%) were published only in Spanish, and 1 (2%) was available in both languages. Most apps (44/49, 90%) were classified in the category of medicine. The other categories were health and well-being (3/49, 6%) and education (2/49, 4%).

Slightly more than half of the apps were free to download (29/49, 59%), whereas the other 41% (20/49) required payment, with a cost ranging from €0.59 (US $0.64) to €179.99 (US $196.10) (median €8.99 [US $9.79]) and a mean cost of €20.82 (US $22.68) (SD €40.81 [US $44.46]). Two apps were for the exclusive use of workers at the center where they were developed, and 1 app could only be used with a code acquired after purchasing a book; therefore, they could not be fully analyzed. In addition, the content of 1 app was unavailable because of a download error that affected the latest versions of Android. In these cases, we collected as much information as possible from the description of the app and images available on the digital distribution platforms.

The average size of the apps was 23.89 (SD 23.28) MB. The content of 27% (13/49) of the apps was updated 6 months before the search. A further 14% (7/49) of the apps were updated in the previous year. A total of 59% (29/49) of the apps had not been updated for more than a year; of these, 12 (24% of the overall apps) had not been updated for more than 3 years. A total of 16% (8/49) apps had not been updated since the date of the first publication. The average time between the date of analysis and the date of the most recent update was 23.3 (SD 23.6) months. iOS apps were excluded from this last analysis because of the lack of information on the day of the most recent update.

About half of the apps were developed by private and for-profit organizations (22/49, 45%) as follows: health-related technology companies (n=12, 24%); non–health-related technology companies (n=9, 18%); and medical publishers (n=1, 2%). A total of 22% (11/49) apps were developed by non–health-related professionals. Among the 78% (38/49) apps developed by health care professionals, 29% (14/49) were developed by individual professionals, whereas the rest were developed by technology companies or medical publishers (13/49, 27%), or with the involvement of a health care organization (eg, hospital, public health agency, or professional society; 11/49, 22%). A complete list of developers is provided in Table 1.

The number of downloads can only be determined in the apps found in the Google Play Store, as this information is not available in the Apple App Store. The median number of downloads was >5000 (range >1 to >100,000). Detailed information regarding the number of downloads is presented in Table 2.

We evaluated the association between the cost of apps and the number of downloads. Owing to the small sample sizes, the number of downloads was broken down for this analysis into 3 categories: 1 to 1000, 1001 to 10,000, and >10,000 downloads. No statistically significant differences were found between the groups (*F*_42_=0.24; *P*=.70).

The analyses of user ratings included 40 apps, as no data were available for 9 apps. The mean overall weighted user rating of apps according to the number of valuations was 4.023 out of 5 (SD 0.71). The average user ratings were almost identical (*t*_38_=−0.01; *P*=.99) for apps developed by health professionals (n=30, mean 4.240, SD 0.707) and non–health professionals (n=10, mean 4.243, SD 0.470). Free apps were rated higher (n=27, mean 4.277, SD 0.680) than paid apps (n=13, mean 4.197, SD 0.621; *t*_38_=−2.27; *P*=.03).

**List of emergency drugs apps and emergency medicine apps.**

**Emergency drugs apps**

- 50 Drugs in emergency
- Antídotos
- Common 50 drugs for emergency
- Drogas en emergencia y UCI
- ED drugs
- Emergency drugs
- Emergency drugs (Antonio Frontera)
- Emergency drugs (Ferrazza)
- Emergency medication reference
- EMS calculator or EMS drugs fast
- EMS drug cards
- Farmacos de urgencias SES or urgencias SES
- FarmaPoniente
- Goteo para vasoactivos
- Guía farmacológica
- Guía URG
- Infusiones
- Medicina de urgencias
- Paramedic drug list
- Perfusiones urgencias
- Pocket drug guide EMS or EMS pocket guide
- UrgRedFasterFH

**Emergency medicine apps**

- AHS EMS MedicalProtocols
- Arritmias urgencias
- Basic emergency care
- Chuletario urgencias extrahospitalarias
- EMAT app
- Emergency central
- Emergency medicine on call
- EMR guide
- EMRA antibiotic guide
- EMRA PressorDex
- EMS ACLS guide
- EMS notes: EMT and paramedic
- EMS pro
- Erres
- ICU
- ICU ER facts made Incred quick
- iTox Urgencias intoxicación
- Manual de procedimientos SAMUR
- Médico de urgencias
- My Emergency Department
- Odonto emergencias
- QuickEM
- URG
- Urgencia HBLT or Guia urgencia HBLT
- Urgencias Extrahospitalarias
- WikEM—Medicina de emergencia
- Zubirán. Manual Terapéutica 7e

**Table 1:** Developers of the apps (N=49).

Developer	Value, n (%)
Individual health professional	14 (29)
Health-related technology company	12 (24)
Non–health-related technology company	9 (18)
Hospital	3 (6)
Public health agency	3 (6)
Individual non–health professional	2 (4)
Medical or pharmaceutical society	2 (4)
Other health professional organization	2 (4)
University	1 (2)
Medical publisher	1 (2)

**Table 2:** Apps classified by the number of downloads (N=43).

Number of downloads	Value, n (%)
1-100	2 (5)
101-1000	5 (12)
1001-5000	12 (28)
5001-10,000	6 (14)
10,001-100,000	12 (28)
>100,000	6 (14)

### Analysis of Contents of Apps

Approximately half of the apps focused on emergency drugs (22/49, 45%), whereas the rest (27/49, 55%) focused on emergency medicine in a broader sense. We did not find statistically significant differences (*z*=−1.7; *P*=.09) between the average user rating of *emergency drugs apps* (4.163/5; 16 apps) and *emergency medicine apps* (4.296/5; 24 apps).

The median number of drugs included in the apps was 66 (range 4 to >5000). The apps classified according to the number of drugs analyzed are shown in Table 3.

Of 49 apps, 6 (12%) analyzed only a specific class of drugs: antidotes (n=2, 33%), vasopressors (n=2, 33%), antibiotics (n=1, 17%), and antiarrhythmics (n=1, 17%).

**Table 3:** Apps classified by number of drugs analyzed (N=47).

Number of drugs	Value, n (%)
1-25	9 (19)
26-50	12 (26)
51-100	10 (21)
101-200	11 (23)
>200	3 (6)
>1000	2 (4)

Table 4 shows the 15 drug characteristics of the apps analyzed. Most apps included information about therapeutic indications (38/48, 79%) and the most common doses (43/49, 88%). Other drug-related concerns found in more than half of the apps were commercial presentations (27/47, 57%), mechanism of action (26/47, 55%), contraindications (26/47, 55%), method of preparation and administration (25/48, 52%), and adverse reactions (24/47, 51%). Only 17% (8/47) of apps provided data on stability and incompatibilities. Identification of high-alert medications was found in 6% (3/47) of the apps. Information on drug costs was present in only 2% (1/47) of the apps. Similarly, information about medication reconciliation in the emergency room was found in only 2% (1/47) of the apps.

Most apps (29/47, 62%) did not include bibliographic references regarding drug-related concerns. The percentage of apps that included this kind of information was 44% (16/36) in the group of apps developed by health professionals and 18% (2/11) in the group of apps developed by non–health professionals (*χ*^2^_1_=2.5; *P*=.12).

**Table 4:** Drug characteristics described in the apps.

Drug characteristic	Value, N	Value, n (%)
Commercial presentations	47	27 (57)
Usual dosage	49	43 (88)
Dose adjustment for renal failure	47	15 (32)
Mechanism of action	47	26 (55)
Therapeutic indications	48	38 (79)
Contraindications	47	26 (55)
Interaction with other medicinal products	47	10 (21)
Use in pregnancy and breastfeeding	47	15 (32)
Adverse reactions	47	24 (51)
Method of preparation and administration	48	25 (52)
Stability data and incompatibilities	47	8 (17)
Identification of high-alert medications	47	3 (6)
Positioning in treatment algorithms	47	19 (40)
Information about reconciliation	47	1 (2)
Cost	47	1 (2)

### Analysis of Drug Information Score

We assigned a score of 0 to 15 according to the number of drug characteristics provided in the app. The mean score was 5.89 (SD 2.91). Of the 47 apps, 22 (47%) apps received a score ranging from 0 to 5, a total of 21 (45%) apps received a score from 6 to 10, and 4 (8%) apps received a score from 11 to 13. There was no correlation between this score and the app user ratings (ρ=−0.17; *P*=.31).

The average score for apps developed by health professionals (n=36, mean 6.00, SD 3.04) was slightly higher than that for apps developed by non–health professionals (n=11, mean 5.55, SD 2.54), although the difference was not significant (*t*_45_=−0.45; *P*=.66). Similarly, no statistically significant differences (*t*_45_=−0.78; *P*=.44) were found between the average score of emergency drugs apps (n=21, mean 5.52, SD 2.75) and emergency medicine apps (n=26, mean 6.19, SD 3.06) or between the average score of free (n=27, mean 5.90, SD 2.91) and paid (n=20, 5.47, SD 3.07) apps (*t*_45_=−0.83; *P*=.40).

Finally, we compared the difference between the number of downloads and drug information score. The average score was 4.57 (SD 1.81) for apps with 1 to 1000 downloads (7/41, 17%), 6.69 (SD 3.28) for apps with 1001 to 10,000 downloads (16/41, 39%), and 6.61 (SD 2.55) for apps with >10,000 downloads (18/41, 44%). No statistically significant differences were found between the groups (*F*_40_=1.63; *P*=.21).

## Discussion

### Overview

Studies on the content of mobile health apps are increasingly frequent, and apps related to relevant diseases such as cancer or COVID-19 infection have recently been analyzed [16,17,23,24]. Nevertheless, research on apps designed for use in emergency rooms remains insufficient. In this study, we provide a comprehensive and unique review of smartphone apps that provide information on drugs for adult emergency care.

The use of mobile devices by emergency health professionals is common, and apps related to this field of medicine are proliferating [10,25]. Emergency rooms are areas where a high volume of patients must be seen within a short period, and work interruptions are very frequent [26]. In this complex environment, incorrect use of mobile devices can increase the risk of distraction and may affect patient safety [9]. Nevertheless, when these devices are used properly, they have enormous potential to improve medical practice, for instance, by allowing quick access to relevant and evidence-based information, which facilitates decision-making and can help reduce error rates. In a recent survey of professionals in an emergency department, most respondents found mobile devices useful for better coordinating care among providers and beneficial for patient care [10].

### Principal Findings on General Characteristics and Comparison With Prior Studies

Our study provides a general perspective on apps designed to help health care professionals with drug management for adult emergency care. Given that medication errors are commonly caused by insufficient information on drugs [12], we analyzed these apps in detail. This is one of the most comprehensive studies of apps aimed at providing information about drugs for health care professionals. Recently, a study identified more than 600 drug-related apps, and approximately two-third of them were categorized within the medication information class [27]. The authors distinguished among apps for patients, apps for health professionals, and apps that can be used by both groups. Recent studies on patient-focused drug apps have analyzed those that help patients understand and take their medications or those with a medication list function [28,29]. In addition, apps for treatment adherence have been the subject of intensive research [30-35]. Some papers have also been published on apps about drug-drug interactions [36,37]. This is an issue traditionally addressed by health care professionals, although nowadays many apps for checking interactions are intended to be used by patients rather than health care professionals.

Knowledge of the characteristics of drug apps designed to be used exclusively by health care professionals is still limited. Few studies have aimed to analyze the functionalities and content of these apps. A study conducted in 2013 identified 306 apps providing drug reference information and prescribing material, and analyzed cost, updates, user ratings, intended area of use, and medical involvement in app development [38]. More recently, a study published in 2017 compared 8 apps for dosage recommendations, adverse reactions, and drug interactions [39]. The quality of the apps targeting medication-related problems has been assessed. Of the 59 apps analyzed, 23 (39%) contained medication information features [40]. Very recently, a study identified 23 drug reference apps with local drug information in Taiwan (including those aimed at both patients and professionals) and analyzed their quality and factors influencing user perceptions [41]. In the field of emergency care, a recent study analyzed apps for the management of drug poisoning [42]. Of the 17 apps identified, 14 (82%) presented diagnosis and treatment guides, and 3 (18%) were specifically on antidotes and their dosage.

In our study, we first collected the information available in the app marketplace descriptions (eg, number of downloads and user ratings) before downloading the apps and analyzing their content in detail. This strategy differs from those of other recent studies, in which a greater number of apps were identified but where the analysis was limited to the marketplace description [8,18,19,38]. Among our main findings, we can highlight that 22% (11/49) of the apps were not developed by health care professionals. This is a lower percentage than that reported in other studies on mobile health apps [18,23,43]. In 2013, there was no evidence of involvement of health care professionals in the development of 32.7% (100/306) of the apps available to support prescribing practice [38]. It should also be noted that apps for patient medication management are developed mainly by the software industry, without the involvement of health care professionals [28,31]. Nevertheless, our findings should be considered relevant, given that the apps we analyzed are intended to be used in complex and emergency situations. In addition, information on authorship is scarce in many of the apps evaluated.

More importantly, we found that more than half of the apps (29/47, 62%) did not include bibliographic references or had not been updated for more than a year (29/49, 59%). Our results are in accordance with a previous study analyzing 23 apps with medication information, most of which did not provide supporting references [40]. Of particular concern is the lack of updates in the apps analyzed in our study, as this indicator has worsened compared with the study conducted by Haffey et al [38], in which 44.4% (136/306) of the apps had either been released or updated within the last 6 months, and a further 24.2% (74/306) within 1 year [38]. These concerns raise doubts about the quality and reliability of the information provided by these apps aimed at emergency health care professionals, as incorrect drug information may remain for long periods.

Doubts arise when a health app is developed by non–health professionals [44,45]. We found that bibliographic references were included in 44% (16/36) of the apps developed by health professionals and in only 18% (2/11) of the apps developed by non–health professionals. This result was not statistically significant (*P*=.12), probably because of the small sample size, although it highlights the uncertainty surrounding the sources of information provided in apps developed by non–health professionals. The reliability and authority of information should be analyzed by health care professionals who are more capable of evaluating, reviewing, and verifying the content of health-related apps. In the field of medication, pharmacists should play a vital role in reviewing apps.

About half of the apps (20/49, 41%) required payment to access all the content, with a cost ranging from €0.59 (US $0.64) to €179.99 (US $196.10). This is a similar percentage than that observed in a recent study on drug poisoning management apps [42]. Nevertheless, it is considerably higher than that observed in other recent reviews of apps for medical emergencies [8], medication management and adherence for patients [28,32], or checking for drug-drug interactions [36]. We hypothesize that these differences could arise because the apps analyzed in our study are aimed exclusively at health care professionals and are designed for use in health care facilities. A study conducted in 2013 on apps to support drug prescribing or provide pharmacology education showed that 68% (208/306) of the apps required payment, with a mean price of £14.25 (US $18.57) per app and a range of £0.62 (US $0.81) to £101.90 (US $132.76) [38]. The cost of apps also seems to be influenced by the origin of the developer [41]. In any case, cost is an important determinant in the decision to adopt a mobile health app, regardless of age group and socioeconomic status [46]. In addition, payment for the apps analyzed in our study could be a relevant limitation for health care professionals who only occasionally work in emergency rooms, as is common in many hospitals.

To date, few studies have analyzed the factors that influence user satisfaction with apps [18,47,48]. The number of downloads and user ratings are usually correlated and have been proposed as indicators of acceptability and satisfaction with mobile health apps [49,50]. A secondary objective of our study was to learn more about user behavior with emergency medicine apps, for which we analyzed whether factors such as cost, the main theme of the app (emergency medicine or emergency drugs), or the app developer (health-related or non–health related) could influence user ratings. The free apps analyzed in our study had higher user ratings than paid apps, although no association was found between the cost and number of downloads. We found no further statistically significant differences, probably because of the small sample size. The number of downloads and user ratings probably depend on multiple factors. Navigation, performance, visual appeal, credibility, and quantity of information have recently been identified as the most influential factors on higher user ratings in a study analyzing 23 drug reference apps [41]. Previous studies have reported highly variable results for the influence of expert involvement in app development on user ratings and the number of downloads [18,41,49]. In any case, user ratings and downloads should not be considered good predictors of the quality and reliability of medical apps because they could be influenced by other factors, such as low price, in-app purchase options, in-app advertisements, and recent updates [18,51,52]. In our study, the number of downloads, cost, and user ratings were not associated with a score created to quantify the variety of relevant information on drug characteristics in the apps (*P*=.21, *P*=.40, and *P*=.31, respectively). Further research should analyze the reliability of the clinical content of drug information apps and corroborate its association with a greater intention to use or better user satisfaction.

### Drug Information Gaps

At present, there are no standardized guidelines for assessing the clinical content and quality of mobile health apps [18]. A highly specific quality assessment tool was developed to assess the quality of apps targeting medication-related problems, including those with medication information features [40]. Nevertheless, the most commonly used methodology to assess the quality of medical apps is the Mobile Application Rating Scale [53-55], as well as in studies on drug apps [30,36,37,41]. The total number of features has been associated with the total Mobile Application Rating Scale score in a study on apps for potential drug-drug interaction decision support [36].

In our study, we paid special attention to the information provided by the apps regarding relevant drug characteristics. We found that most apps included information about the usual dosage (43/49, 88%) and therapeutic indications (38/48, 79%). Nevertheless, other relevant characteristics were found in less than half of the apps, such as dose adjustment for renal failure (15/47, 32%) and use in pregnancy and breastfeeding (15/47, 32%). Interaction with other medicinal products was found in only 21% (10/47) of the apps, despite being a major problem in patient safety. Drug-drug interaction checks are one of the most frequent functional categories within the current medication-related app landscape [27,56], but relevant quality and accuracy problems have been detected in apps, including this feature [36,37]. In addition, other relevant information on drug safety, such as contraindications (26/47, 55%) and adverse reactions (24/47, 51%), was found in approximately half of the apps analyzed in our study. Furthermore, it is worrying that only 6% (3/47) of the apps clearly identified high-alert medications, despite efforts made to avoid errors with these drugs [12].

In clinical practice, many medication-related inquiries are about the method of administration; however, our study showed that this information is included in slightly more than half of the apps (25/48, 52%). In addition, stability data and incompatibilities were present in only 17% (8/47) of the apps. Nurses have also been reported to be frequent app users in daily practice, albeit at a slightly lower percentage than that observed by physicians [10]. Therefore, apps for the use of drugs in the emergency department should be designed to provide more information on drug administration characteristics.

Finally, incorrect medication reconciliation in the emergency department can lead to relevant medication errors [57]. We found only 1 app that appropriately addressed this issue, including information on the maximum time to carry out reconciliation or the possible presence of withdrawal syndrome. Given that medication reconciliation has been considered the most relevant activity carried out by pharmacists in emergency departments [58], it would be desirable for apps related to emergency drugs to provide more information on this matter.

### Recommendations for Development of an Emergency Drugs App

There are a growing number of health apps on the market with highly variable designs and content, and it is difficult to determine which are the most useful for health care professionals. Given the relative absence of legislation on medical apps [59] and the risks associated with drugs used in the emergency room, it would be interesting to propose a series of improvements in the content of apps for emergency drugs. The results of our study and clinical experience enable us to make several recommendations.

Design, ease of use, and the ability to quickly respond to questions that arise during daily clinical practice are especially relevant characteristics, considering that these apps are to be used in a stressful environment. The success of an app for emergency professionals depends on quickly obtaining a reliable response.

Our findings could help developers design apps that provide drug-related information most frequently demanded by health care professionals. Drug information centers have historically received the most inquiries regarding therapeutic indications, adverse reactions, and identification of medical products [13]. In addition, information on contraindications, appropriate dosage, and major drug-drug interactions should be included to prevent major adverse events [11]. We provided a score to measure the amount of drug information included in each app, and our results showed that a greater amount of information is not necessarily associated with better user ratings. Therefore, it could be beneficial to design apps with content aimed exclusively at doctors and apps for nurses, although with maximum information of interest for each of these professionals. For example, apps with information on drug administration and incompatibilities would have the potential to help nursing staff by reducing their workload and, ultimately, the risk of drug-related errors. Strategies to identify high-alert medications should be included in all emergency drug apps, regardless of the group of health care professionals they focus on [12].

We recommend caution with respect to the sources of information used to elaborate the content of the app to ensure that it is reliable. Apps should only be considered reliable based on an extensive literature review, expert panel review, or peer review. The author’s affiliation and bibliographic references to scientific and clinical evidence should always be clearly shown [60], and health professionals participating in reviewing and app updates should be clearly identified.

As previously suggested [16], we believe that future legislation should require a more comprehensive description of the mobile app marketplace, with detailed information on authorship and the process used to review app functionalities and the clinical information provided. All information must be supported by appropriate bibliographic references, and developers should preferably be clinicians with experience writing or synthesizing medical evidence. Thus, the information provided, which should be checked by independent reviewers or endorsed by health organizations of recognized prestige, will be more reliable. In addition, we suggest that app developers clearly identify the target user group and provide the maximum amount of drug information relevant to each professional category. It may also be relevant for a partner with a technology company to make apps more attractive and user-friendly.

### Limitations and Future Research Directions

First, our study was limited by the inclusion criteria. There are hundreds (perhaps thousands) of apps providing drug-related information, and some may be useful for emergency room professionals; however, they were not analyzed in this study because our aim was to review apps specifically related to emergency drugs or medicine in adults. Drug information indicators were selected and analyzed by the authors and were therefore not validated. A more comprehensive analysis of drug information apps may be the subject of future research, for which our methodology could prove useful. Our approach could be adapted to analyze apps related to child health care or to include indicators not described in our study, such as information on pharmacokinetic properties, therapeutic drug monitoring, and pharmacogenomics. Other limitations are associated with the study design. We only analyzed the Android version when the same app was available on Android and iOS platforms. It should be noted that some characteristics, such as the date of the last update, may vary among platforms. In addition, we analyzed apps in English and Spanish. Although Spanish is the language with the second highest number of native speakers, many health professionals are not sufficiently competent in the language to use these apps comfortably. Our study was also limited by the fact that it only analyzed whether a series of drug characteristics of interest were included in the app. Further research is needed to evaluate the clinical accuracy of the drug information provided by the apps. One possible approach would be a peer-review process to evaluate app contents in terms of the reliability and quality of information according to the best available clinical evidence.

### Conclusions

We conducted a comprehensive and unique systematic review of apps that provide information on drugs for adult emergency care. We identified 49 apps according to the PRISMA methodology and conducted a content analysis on most of them. Health-related app developers, the main topic of the app (emergency drugs or emergency medicine), and a greater amount of drug information were not associated with higher app user ratings. Slightly less than half of the apps (20/49, 41%) required payment, with a cost ranging from €0.59 (US $0.64) to €179.99 (US $196.10). We noted that 22% (11/49) of the apps were not developed by health care professionals. Most apps include information about the usual dosage and therapeutic indications, although information on safety and drug administration is much less frequent. Very few apps provide relevant information, such as high-alert medication notices and instructions for drug reconciliation. In addition, more than half of the apps (29/47, 62%) did not include bibliographic references. These findings cast doubts on the quality of many apps. Therefore, we propose a series of issues that should be considered when developing an app of these characteristics and advocate for greater regulation and more frequent and documented review of app content.

## Abbreviations

PRISMA: Preferred Reporting Items for Systematic Reviews and Meta-Analyses

## References

[1] (2017). mHealth: use of appropriate digital technologies for public health: report by the Director-General. World Health Organization.

[2] Number of mHealth apps available at Google Play and App Store from 1st quarter 2015 to 3rd quarter 2020. Statista.

[3] Alonso SG, Marques G, Barrachina I, Garcia-Zapirain B, Arambarri J, Salvador JC, de la Torre Díez I (2021). Telemedicine and e-Health research solutions in literature for combatting COVID-19: a systematic review. Health Technol (Berl).

[4] de la Torre Díez I, Alonso SG, Hamrioui S, López-Coronado M, Cruz EM (2018). Systematic review about QoS and QoE in telemedicine and eHealth services and applications. J Med Syst.

[5] Emergency department visits. Centers for Disease Control and Prevention.

[6] Informe Anual del Sistema Nacional de Salud 2018 Resumen ejecutivo. Ministerio de Sanidad.

[7] Farmer B (2016). Patient safety in the emergency department. Emerg Med.

[8] Plaza Roncero A, Marques G, Sainz-De-Abajo B, Martín-Rodríguez F, Del Pozo Vegas C, Garcia-Zapirain B, de la Torre-Díez I (2020). Mobile health apps for medical emergencies: systematic review. JMIR Mhealth Uhealth.

[9] Alameddine M, Soueidan H, Makki M, Tamim H, Hitti E (2019). The use of smart devices by care providers in emergency departments: cross-sectional survey design. JMIR Mhealth Uhealth.

[10] Hitti E, Hadid D, Melki J, Kaddoura R, Alameddine M (2021). Mobile device use among emergency department healthcare professionals: prevalence, utilization and attitudes. Sci Rep.

[11] Hammond DA, Gurnani PK, Flannery AH, Smetana KS, Westrick JC, Lat I, Rech MA (2019). Scoping review of interventions associated with cost avoidance able to be performed in the intensive care unit and emergency department. Pharmacotherapy.

[12] Ortmann MJ, Johnson EG, Jarrell DH, Bilhimer M, Hayes BD, Mishler A, Pugliese RS, Roberson TA, Slocum G, Smith AP, Yabut K, Zimmerman DE (2021). ASHP guidelines on emergency medicine pharmacist services. Am J Health Syst Pharm.

[13] Rosenberg JM, Koumis T, Nathan JP, Cicero LA, McGuire H (2004). Current status of pharmacist-operated drug information centers in the United States. Am J Health Syst Pharm.

[14] Moher D, Liberati A, Tetzlaff J, Altman DG, PRISMA Group (2009). Preferred reporting items for systematic reviews and meta-analyses: the PRISMA statement. Ann Intern Med.

[15] Mobile operating system market share worldwide. Statcounter Global Stats.

[16] Charbonneau DH, Hightower S, Katz A, Zhang K, Abrams J, Senft N, Beebe-Dimmer JL, Heath E, Eaton T, Thompson HS (2020). Smartphone apps for cancer: a content analysis of the digital health marketplace. Digit Health.

[17] Collado-Borrell R, Escudero-Vilaplana V, Villanueva-Bueno C, Herranz-Alonso A, Sanjurjo-Saez M (2020). Features and functionalities of smartphone apps related to COVID-19: systematic search in app stores and content analysis. J Med Internet Res.

[18] Biviji R, Vest JR, Dixon BE, Cullen T, Harle CA (2020). Factors related to user ratings and user downloads of mobile apps for maternal and infant health: cross-sectional study. JMIR Mhealth Uhealth.

[19] Aydin G, Silahtaroglu G (2021). Insights into mobile health application market via a content analysis of marketplace data with machine learning. PLoS One.

[20] High-alert medications in acute care settings. Institute for Safe Medication Practices.

[21] Assuring medication accuracy at transitions in care: medication reconciliation. The High 5s Project.

[22] Freelon D (2010). ReCal: intercoder reliability calculation as a web service. Int J Internet Sci.

[23] Collado-Borrell R, Escudero-Vilaplana V, Ribed-Sánchez A, Ibáñez-García S, Herranz-Alonso A, Sanjurjo-Sáez M (2016). Smartphone applications for cancer patients; what we know about them?. Farm Hosp.

[24] Kondylakis H, Katehakis DG, Kouroubali A, Logothetidis F, Triantafyllidis A, Kalamaras I, Votis K, Tzovaras D (2020). COVID-19 mobile apps: a systematic review of the literature. J Med Internet Res.

[25] Gaziel-Yablowitz M, Schwartz DG (2019). A review and assessment framework for mobile-based emergency intervention apps. ACM Comput Surv.

[26] Chisholm CD, Dornfeld AM, Nelson DR, Cordell WH (2001). Work interrupted: a comparison of workplace interruptions in emergency departments and primary care offices. Ann Emerg Med.

[27] Degelsegger-Márquez A, Trunner K, Piso B (2020). Medication apps - a systematic search and classification. Stud Health Technol Inform.

[28] Tabi K, Randhawa AS, Choi F, Mithani Z, Albers F, Schnieder M, Nikoo M, Vigo D, Jang K, Demlova R, Krausz M (2019). Mobile apps for medication management: review and analysis. JMIR Mhealth Uhealth.

[29] Diaz-Skeete YM, McQuaid D, Akinosun AS, Ekerete I, Carragher N, Carragher L (2021). Analysis of apps with a medication list functionality for older adults with heart failure using the mobile app rating scale and the IMS institute for healthcare informatics functionality score: evaluation study. JMIR Mhealth Uhealth.

[30] Santo K, Richtering SS, Chalmers J, Thiagalingam A, Chow CK, Redfern J (2016). Mobile phone apps to improve medication adherence: a systematic stepwise process to identify high-quality apps. JMIR Mhealth Uhealth.

[31] Ahmed I, Ahmad NS, Ali S, Ali S, George A, Saleem Danish H, Uppal E, Soo J, Mobasheri MH, King D, Cox B, Darzi A (2018). Medication adherence apps: review and content analysis. JMIR Mhealth Uhealth.

[32] Park JY, Li J, Howren A, Tsao NW, De Vera M (2019). Mobile phone apps targeting medication adherence: quality assessment and content analysis of user reviews. JMIR Mhealth Uhealth.

[33] Pérez-Jover V, Sala-González M, Guilabert M, Mira JJ (2019). Mobile apps for increasing treatment adherence: systematic review. J Med Internet Res.

[34] Armitage LC, Kassavou A, Sutton S (2020). Do mobile device apps designed to support medication adherence demonstrate efficacy? A systematic review of randomised controlled trials, with meta-analysis. BMJ Open.

[35] Al-Arkee S, Mason J, Lane DA, Fabritz L, Chua W, Haque MS, Jalal Z (2021). Mobile apps to improve medication adherence in cardiovascular disease: systematic review and meta-analysis. J Med Internet Res.

[36] Kim BY, Sharafoddini A, Tran N, Wen EY, Lee J (2018). Consumer mobile apps for potential drug-drug interaction check: systematic review and content analysis using the mobile app rating scale (MARS). JMIR Mhealth Uhealth.

[37] Shen C, Jiang B, Yang Q, Wang C, Lu KZ, Gu M, Yuan J (2021). Mobile apps for drug-drug interaction checks in chinese app stores: systematic review and content analysis. JMIR Mhealth Uhealth.

[38] Haffey F, Brady RR, Maxwell S (2014). Smartphone apps to support hospital prescribing and pharmacology education: a review of current provision. Br J Clin Pharmacol.

[39] Apidi NA, Murugiah MK, Muthuveloo R, Soh YC, Caruso V, Patel R, Ming LC (2017). Mobile medical applications for dosage recommendation, drug adverse reaction, and drug interaction: review and comparison. Ther Innov Regul Sci.

[40] Loy JS, Ali EE, Yap KY (2016). Quality assessment of medical apps that target medication-related problems. J Manag Care Spec Pharm.

[41] Chen Y, Liao W, Su M, Lin Y (2021). Personalized and self-management: systematic search and evaluation quality factors and user preference of drug reference apps in Taiwan. J Pers Med.

[42] Hajesmaeel Gohari S, Bahaadinbeigy K, Tajoddini S, R Niakan Kalhori S (2020). Drug poisoning management using smartphones: an apps review study based on use case classification model. BMJ Innov.

[43] Subhi Y, Bube SH, Rolskov Bojsen S, Skou Thomsen AS, Konge L (2015). Expert involvement and adherence to medical evidence in medical mobile phone apps: a systematic review. JMIR Mhealth Uhealth.

[44] van Velsen L, Beaujean DJ, van Gemert-Pijnen JE (2013). Why mobile health app overload drives us crazy, and how to restore the sanity. BMC Med Inform Decis Mak.

[45] Tomlinson M, Rotheram-Borus MJ, Swartz L, Tsai AC (2013). Scaling up mHealth: where is the evidence?. PLoS Med.

[46] Peng W, Kanthawala S, Yuan S, Hussain SA (2016). A qualitative study of user perceptions of mobile health apps. BMC Public Health.

[47] Mendiola MF, Kalnicki M, Lindenauer S (2015). Valuable features in mobile health apps for patients and consumers: content analysis of apps and user ratings. JMIR Mhealth Uhealth.

[48] Carlo AD, Hosseini Ghomi R, Renn BN, Areán PA (2019). By the numbers: ratings and utilization of behavioral health mobile applications. NPJ Digit Med.

[49] Pereira-Azevedo N, Osório L, Cavadas V, Fraga A, Carrasquinho E, Cardoso de Oliveira E, Castelo-Branco M, Roobol MJ (2016). Expert involvement predicts mHealth app downloads: multivariate regression analysis of urology apps. JMIR Mhealth Uhealth.

[50] Huang H, Bashir M (2017). Users' adoption of mental health apps: examining the impact of information cues. JMIR Mhealth Uhealth.

[51] Ghose A, Han SP (2014). Estimating demand for mobile applications in the new economy. Manag Sci.

[52] Plante TB, O'Kelly AC, Macfarlane ZT, Urrea B, Appel LJ, Miller Iii ER, Blumenthal RS, Martin SS (2018). Trends in user ratings and reviews of a popular yet inaccurate blood pressure-measuring smartphone app. J Am Med Inform Assoc.

[53] Stoyanov SR, Hides L, Kavanagh DJ, Zelenko O, Tjondronegoro D, Mani M (2015). Mobile app rating scale: a new tool for assessing the quality of health mobile apps. JMIR Mhealth Uhealth.

[54] Amor-García MÁ, Collado-Borrell R, Escudero-Vilaplana V, Melgarejo-Ortuño A, Herranz-Alonso A, Arija A, Sanjurjo-Sáez M (2020). Assessing apps for patients with genitourinary tumors using the mobile application rating scale (MARS): systematic search in app stores and content analysis. JMIR Mhealth Uhealth.

[55] Woulfe F, Fadahunsi KP, Smith S, Chirambo GB, Larsson E, Henn P, Mawkin M, O' Donoghue J (2021). Identification and evaluation of methodologies to assess the quality of mobile health apps in high-, low-, and middle-income countries: rapid review. JMIR Mhealth Uhealth.

[56] Hammar T, Hamqvist S, Zetterholm M, Jokela P, Ferati M (2021). Current knowledge about providing drug-drug interaction services for patients-a scoping review. Pharmacy (Basel).

[57] Santell JP (2006). Reconciliation failures lead to medication errors. Jt Comm J Qual Patient Saf.

[58] García-Martín Á, Maroun-Eid C, Campino-Villegas A, Oliva-Manuel B, Herrero-Ambrosio A, Quintana-Díaz M (2017). Perception survey on the value of the hospital pharmacist at the emergency department. Farm Hosp.

[59] Gordon WJ, Landman A, Zhang H, Bates DW (2020). Beyond validation: getting health apps into clinical practice. NPJ Digit Med.

[60] Health on the net foundation. HONcode principles.

